# Variations in Stress Sensitivity and Genomic Expression in Diverse *S. cerevisiae* Isolates

**DOI:** 10.1371/journal.pgen.1000223

**Published:** 2008-10-17

**Authors:** Daniel J. Kvitek, Jessica L. Will, Audrey P. Gasch

**Affiliations:** 1 Laboratory of Genetics, University of Wisconsin–Madison, Madison, Wisconsin, United States of America; 2 Genome Center of Wisconsin, University of Wisconsin–Madison, Madison, Wisconsin, United States of America; University of Oxford, United Kingdom

## Abstract

Interactions between an organism and its environment can significantly influence phenotypic evolution. A first step toward understanding this process is to characterize phenotypic diversity within and between populations. We explored the phenotypic variation in stress sensitivity and genomic expression in a large panel of *Saccharomyces* strains collected from diverse environments. We measured the sensitivity of 52 strains to 14 environmental conditions, compared genomic expression in 18 strains, and identified gene copy-number variations in six of these isolates. Our results demonstrate a large degree of phenotypic variation in stress sensitivity and gene expression. Analysis of these datasets reveals relationships between strains from similar niches, suggests common and unique features of yeast habitats, and implicates genes whose variable expression is linked to stress resistance. Using a simple metric to suggest cases of selection, we found that strains collected from oak exudates are phenotypically more similar than expected based on their genetic diversity, while sake and vineyard isolates display more diverse phenotypes than expected under a neutral model. We also show that the laboratory strain S288c is phenotypically distinct from all of the other strains studied here, in terms of stress sensitivity, gene expression, Ty copy number, mitochondrial content, and gene-dosage control. These results highlight the value of understanding the genetic basis of phenotypic variation and raise caution about using laboratory strains for comparative genomics.

## Author Summary

Much attention has been given to the ways in which organisms evolve new phenotypes and the influence of the environment on this process. A major focus of study is defining the genetic basis for phenotypes important for organismal fitness. As a first step toward this goal, we surveyed phenotypic variation in diverse yeast strains collected from different environments by characterizing variations in stress resistance and genomic expression. We uncovered many phenotypic differences across yeast strains, both in stress tolerance and gene expression. The similarities and differences of the strains analyzed uncovered phenotypes shared by strains that live in similar environments, suggesting common features of yeast niches as well as mechanisms that different strains use to thrive in those conditions. We provide evidence that some characteristics of strains isolated from oak tree soil have been selected for, perhaps because of the shared selective pressures imposed by their environment. One theme emerging from our studies is that the laboratory strain of yeast, long used as a model for yeast physiology and basic biology, is aberrant compared to all other strains. This result raises caution about making general conclusions about yeast biology based on a single strain with a specific genetic makeup.

## Introduction

A major focus of genetic study is to elucidate the effects of genetic variation on phenotypic diversity. The evolution of phenotypes is often driven by environmental factors and the interactions between each organism and its environment. Recently, there has been a renewed interest in characterizing the diversity and ecology of organisms long used in the laboratory as models for biological study. Yeast, worms, flies, and mice have been studied on a molecular level for decades and have provided many insights into basic biology. However, most of our knowledge base exists for only a handful of domesticated lines. Little is known about the natural ecology of these organisms or the degree to which individuals of each species vary within and between natural populations.

The budding yeast *Saccharomyces cerevisiae* exists in diverse niches across the world and can be found in natural habitats associated with fruits, tree soil, and insects, in connection with human societies (namely through brewing and baking), and in facultative infections of immuno-compromised individuals [1]. These yeasts are transported by insect vectors and likely through association with human societies. Recent population-genetic studies have begun to explore the genetic diversity of *S. cerevisiae* strains [2]–[5]. These studies have demonstrated little geographic structure in natural yeast populations and relatively low sequence diversity, particularly within vineyard strains. It has been proposed that low sequence diversity in this species may be due to a more recent common ancestor compared to other yeasts [6]. Genomic comparisons also suggest low rates of outcrossing between strains [7], which may limit the fixation of genetic differences under selection by reducing effective population sizes [8].

Although the genetic diversity of *S. cerevisiae* populations is emerging from large-scale sequencing projects, the phenotypic diversity within and between yeast populations has been less systematically studied. Myriad studies have characterized strain-specific differences in specific phenotypes to identify the genetic basis for phenotypes of interest (for example, those related to wine making [9], thermotolerance [10]–[12], sporulation efficiency [13]–[16], drug sensitivity [17]–[19], and others [20]–[25]). The degree to which these phenotypes vary across diverse strains has not been systematically explored. Other genomic studies have investigated variation in genomic expression across strains, with the goal of investigating the mode and consequence of gene-expression evolution [26]–[30]. These studies demonstrated significant variation in gene expression between strains, and in some cases pointed to the genetic basis for those differences [27], [31]–[35]. However, each study investigated only a few strains, typically vineyard strains. The broader phenotypic variation across diverse yeast strains and populations, particularly natural isolates, is largely uncharacterized.

Here we investigated the variation in stress sensitivity and genomic expression in a large panel of *Saccharomyces* strains. We quantified the sensitivity of 52 strains collected from diverse niches to 14 environmental conditions and measured genomic expression in 18 of these strains growing in standard medium. We observe a large amount of phenotypic variation, both in terms of stress sensitivity and gene expression. Associations among phenotypes revealed relationships between environmental conditions and among yeast strains. One case in particular suggests that genetically diverse strains collected from oak soil have undergone selection for growth in a common niche. This study provides a representative description of expression variation and stress sensitivity within and across yeast populations, particularly non-laboratory strains, setting the stage for elucidating the genetic basis of this variation.

## Results

### Variation in Environmental Sensitivity in a Large Panel of *Saccharomyces* Strains

**Figure 1:**
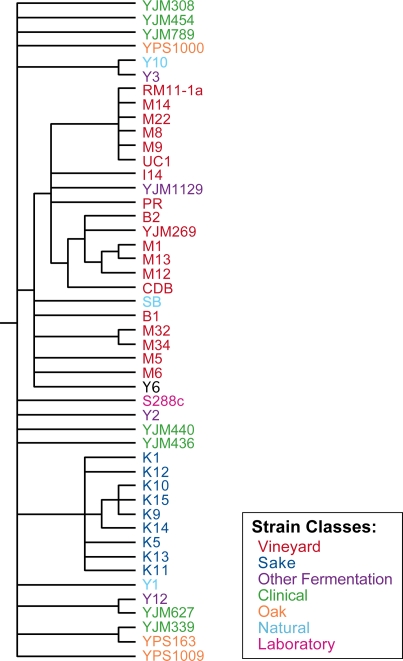
Phylogeny of *Saccharomyces* strains used in this study. The phylogeny was inferred from 13,061 bp of coding and non-coding sequence generated by [2] and this study, using the program MrBayes [73]. Nodes with a posterior probability<0.9 are collapsed. Strains are color coded according to the niche from which they were originally isolated, as shown in the key at the bottom of the figure.

Fay and Benavides conducted a population-genetic study of 81 *Saccharomyces* strains by analyzing ∼7 kb of coding and non-coding sequence from each isolate [2]. We characterized the phenotypic diversity of 52 of these strains, shown in Figure 1. This set included natural isolates from European vineyards, yeasts collected from African palm-wine fermentations, commercial wine- and sake-producing strains, clinical yeasts, natural isolates collected from African and Asian fruit substrates, strains from oak-tree soil and exudates from the Northeastern United States, three common lab strains, and other isolates (see Table S1 and [2] for references). We also characterized two haploid *S. cerevisiae* strains (RM11-1a and YJM789) and three other *Saccharomyces* species (*S. paradoxus*, *S. mikatae*, and *S. bayanus*) for which whole-genome sequence is available [36],[37]. Each strain was grown under 31 different conditions representing 14 unique environments, chosen to provoke diverse physiological responses. These environments varied in nutrient composition, growth temperature, and presence of toxic drugs, heavy metals, oxidizing agents, and osmotic/ionic stress. Cells were grown on solid medium in the presence of each environmental variable, and viability was scored relative to a no-stress control for each strain (see Materials and Methods for details).

**Figure 2:**
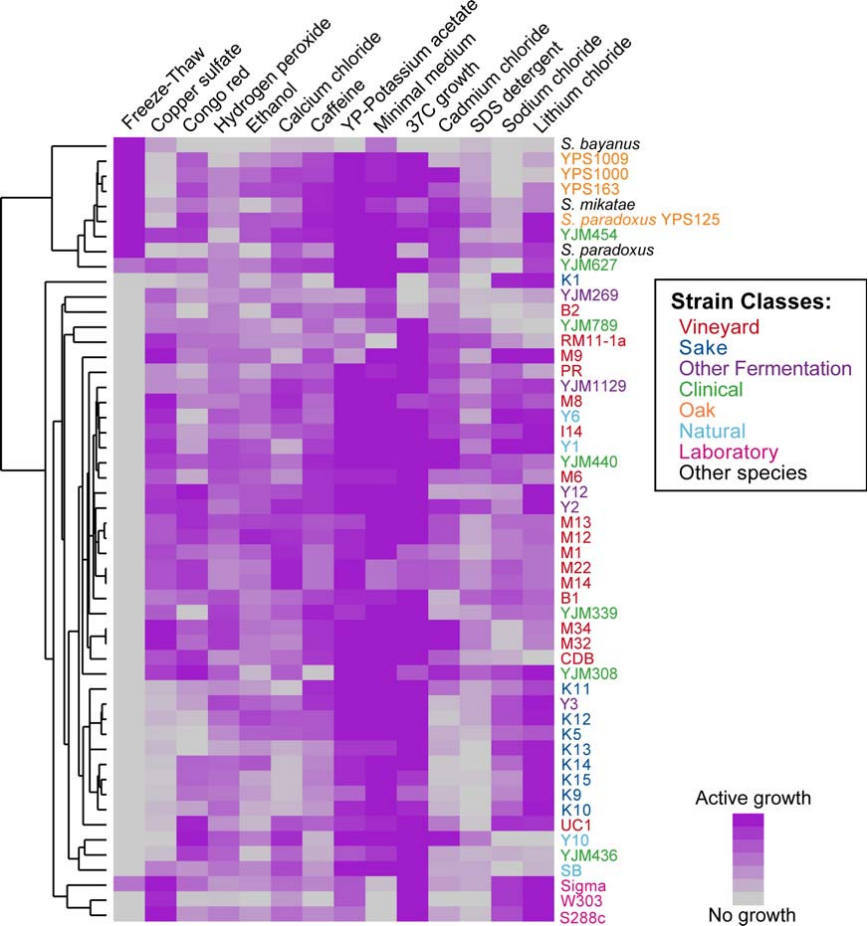
Phenotypic variation in diverse *Saccharomyces* strains. The viability of 52 *Saccharomyces* strains and species grown under 14 different environmental conditions was measured. Strains were grown in at least duplicate on solid agar plates containing 1–3 doses of each environmental variable, as described in Materials and Methods. Each row on the plot represents a different strain and each column indicates a given environment. Colored boxes represent the average growth score of each strain grown in each environment, according to the key shown at the lower right. Strains and conditions were organized by hierarchical clustering using the Pearson correlation as a similarity metric.

The results reveal a tremendous amount of phenotypic diversity in environmental sensitivity (Figure 2). Although there were similarities between strains, no two strains were exactly alike in phenotypic profile. Each displayed a propensity for growth under at least one environment and sensitivity to one or more conditions. Some strains were generally tolerant to stressful environments across the board. For example, strain Y2, originally collected from a Trinidadian rum distillery, and clinical isolates YJM454 and YJM440 were tolerant of most of these conditions, while the *S. bayanus* strain used in our study was sensitive to nearly all stresses tested. Several strains, including commercial sake-producing strains, showed a wide standard deviation of growth scores across the stresses, reflecting that they were either highly sensitive or highly resistant to different stresses. In contrast, most vineyard isolates grew moderately well in most of the environments examined (see Discussion).

Exploration of the range of strain sensitivities measured for each environment also suggested common and unique features of *Saccharomyces*' habitats. Collectively, this set of strains showed the greatest variation in copper sulfate tolerance, sodium chloride resistance, and freeze-thaw survival, implicating these as niche-specific features not generally experienced by yeast. In contrast, strains showed the least variation (but some variability nonetheless) for growth on non-fermentable acetate, in minimal medium lacking supplemental amino acids, and at 37°C. Presumably, defects in respiration, prototrophy, and growth at physiological temperature represent a significant selective disadvantage, regardless of the particular niche.

### Strains from Similar Niches Display Similar Profiles of Stress Sensitivity

Hierarchical clustering of the phenotype data revealed interesting relationships between groups of strains. In particular, several groups of strains displayed similar profiles of stress sensitivity across the environments tested (Figure 2). As a group, the sake-producing strains were extremely resistant to lithium chloride but sensitive to copper sulfate, calcium chloride, cadmium chloride, and SDS detergent (p<0.005 based on 10,000 permutations, see Materials and Methods); indeed, this group was slightly more sensitive to stress in general. Many of the vineyard strains shared specific phenotypes, including resistance to copper sulfate, as previously noted for other vineyard strains [26],[38],[29]. The group of laboratory strains was also highly resistant to copper sulfate as well as sodium and lithium chloride. In contrast, strains collected from oak soil were particularly sensitive to copper sulfate and sodium chloride but highly resistant to freeze-thaw stress (p<0.005, 10,000 permutations).

The similarities in phenotypic profiles could arise through selection (either directional or purifying) due to shared selective pressures across strains living in the same environment. Alternatively, phenotypic similarity could result simply if the strains are genetically related due to a recent common ancestor. For example, many of the lab strains are closely related, since a large fraction of their genomes is derived from a common progenitor [39],[40]. We wished to distinguish between these possibilities for other strain groups. Natural selection can be inferred by comparing the population genetic structure (F_ST_) to an analogous measure of phenotypic structure (Q_ST_) [41],[42]. A deviation from unity suggests that either divergent (Q_ST_/F_ST_>1) or purifying (Q_ST_/F_ST_<1) selection has occurred across populations. We wished to analyze each subpopulation separately, and therefore we devised a simple alternative approach to identify deviations from neutral phenotypic variation. We calculated the average pairwise phenotypic distance over the average pairwise genetic distance for pairs of strains collected from the same environment (‘sake’, ‘vineyard’, ‘oak’, ‘clinical’, ‘natural’ or ‘other fermentation’). This ratio was compared to the ratio of distances calculated for pairs of strains between niche groups, generating the parameter P/G. A P/G ratio = 1 is expected under neutrality, where the phenotypic to genetic distance is equal for within-group versus between-group comparisons. In contrast, a value of P/G<1 suggests that the strains within the group are more similar in phenotype than would be expected under the neutral model, whereas a ratio >1 indicates that the strains are phenotypically more variable than expected based on their genetic relatedness.

The results provide evidence of both selection and shared ancestry for different groups of strains. First, the P/G ratio did not deviate significantly from unity for strains in the ‘clinical’, ‘natural’, or ‘other fermentation’ groups (average P/G = 1.02+/−0.22), nor did it deviate significantly for randomized simulations (data not shown). In contrast, P/G was 4.2 and 3.0 for sake strains and vineyard strains, respectively. Thus, the similarity in their phenotypes likely arises due to their recent divergence from a common ancestor. Interestingly, these P/G values were significantly higher than expected by chance (p<0.0001 from 10,000 permutations), suggesting that the strains show *more* phenotypic variation than expected. This could arise if strains have experienced diversifying selection for disparate phenotypes, although it could also result if genetic distances are underrepresented or skewed due to limited sequence data.

In contrast, strains collected from oak-tree exudates and soil are phenotypically more similar than would be expected under a neutral model. We observed a P/G ratio of 0.31 (p = 0.0013 from 10,000 permutations), indicating that phenotypic variation within this group is lower than expected based on the strains' genetic relatedness. This suggests that the strains have undergone selection for growth in a common environment (see Discussion). Consistent with this model, the *S. paradoxus* strain YPS125, also collected from Northeastern oak flux [6], is phenotypically more similar to *S. cerevisiae* strains collected from that environment (pairwise R of 0.61, 0.66, and 0.77 to YPS1000, YPS1009, and YPS163, respectively) than to the other *S. paradoxus* strain in our collection (R = 0.51). At least some of the phenotypes shared by these strains are likely important for their ability to thrive in their niche (see Discussion).

### Extensive Variation in Genomic Expression in Non-Laboratory Strains

Numerous studies have characterized differences in genomic expression between individual strains of yeast, typically vineyard and lab strains [13], [26]–[31],[34],[43]. To more broadly survey the variation in genomic expression across populations, we measured whole-genome expression in 17 non-laboratory strains compared to that in the diploid S288c-derived strain DBY8268, using 70mer oligonucleotide arrays designed against the S288c genome. The long oligos used to probe each gene minimize hybridization defects due to sequence differences from S288c. We verified this by hybridizing genomic DNA from 6 strains of varying genetic distance from S288c: indeed, fewer than 5% of the observed gene expression differences described below could be explained by defective hybridization to the arrays (see Materials and Methods). Therefore the vast majority of measured expression differences are due to differences in transcript abundance.

**Figure 3:**
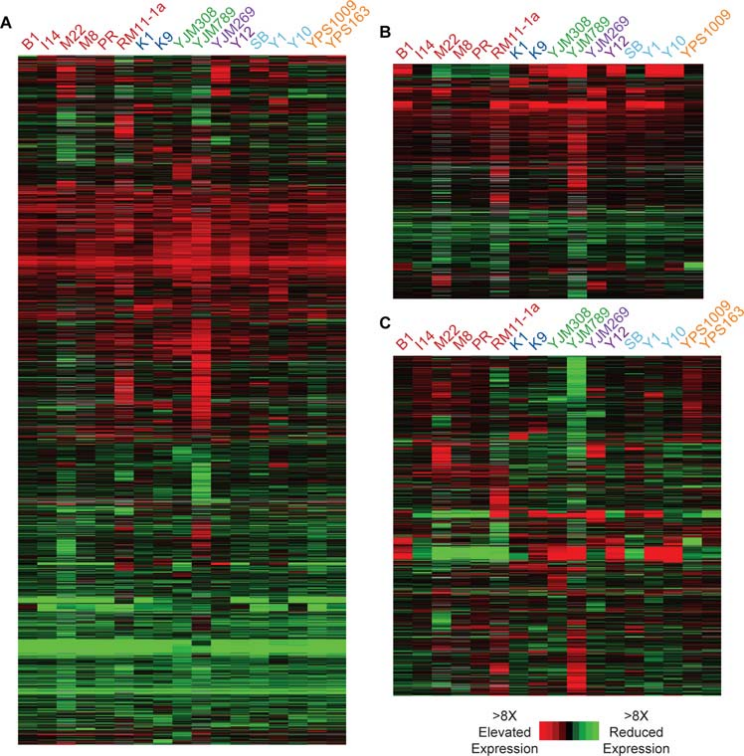
Variation in gene expression in *S. cerevisiae* isolates. The diagrams show the average log2 expression differences measured in the denoted strains. Each row represents a given gene and each column represents a different strain, color-coded as described in Figure 1. (A) Expression patterns of 2,680 genes that varied significantly (FDR = 0.01, paired t-test) in at least one strain compared to S288c. (B) Expression patterns of 953 genes that varied significantly in at least one strain compared to strain YPS163 (FDR = 0.01, unpaired t-test). For (A) and (B), a red color indicates higher expression and a green color represents lower expression in the denoted strain compared to S288c, according to the key. (C) Expression patterns of 1,330 genes that varied significantly (FDR = 0.01, paired t-test) in at least one strain compared to the mean expression of all 17 strains. Here, red and green correspond to higher and lower expression, respectively, compared to the mean expression of that gene in all strains. Genes were organized independently in each plot by hierarchical clustering.

A striking number of yeast genes showed differential expression from the laboratory strain in at least one other strain (Figure 3A). Of the ∼5,700 predicted *S. cerevisiae* open reading frames, 2680 (∼47%) were statistically significantly altered in expression (false discovery rate, FDR = 0.01) in at least one non-laboratory strain compared to S288c, with an average of 480 genes per strain. At an FDR of 0.05, over 70% of genes were significantly altered in expression in at least one non-lab strain (Table 1). The number of expression differences is comparable to that observed by Brem et al., who reported over half of yeast genes differentially expressed between the vineyard strain RM11-1a and S288c [27].

**Table 1:** Number of differentially expressed genes in 17 non-laboratory strains.

Strain	Expression Differences from S288c	FDR 0.01	
		Expression Differences from Mean	Expression Differences from YPS163
B1	98 a, 176 b (4.6)	33, 18 (0.085)	84, 25 (1.8)
I14	112, 260 (6.2)	14, 17 (0.5)	15, 14 (0.5)
K1	174, 239 (6.9)	59, 32 (1.5)	63, 22 (1.4)
K9	232, 212 (7.5)	70, 33 (1.7)	100, 22 (2.1)
M22	220, 550 (15)	103, 245 (6.8)	75, 69 (2.8)
M8	133, 311 (7.5)	10, 80 (1.5)	13, 18 (0.5)
PR	160, 271 (7.4)	9, 73 (1.4)	11, 13 (0.4)
RM11-1a	326, 253 (11.4)	191, 116 (6.1)	151, 53 (4)
SB	131, 272 (6.9)	24, 31 (0.9)	63, 21 (1.4)
Y1	185, 189 (6.4)	92, 14 (1.8)	92, 18 (1.9)
Y10	120, 263 (6.5)	74, 20 (1.6)	59, 11 (1.2)
Y12	162, 195 (5.9)	36, 14 (0.8)	46, 9 (0.9)
YJM269	285, 255 (8.9)	148, 53 (3.3)	132, 43 (2.9)
YJM308	364, 288 (11)	153, 34 (3.1)	142, 40 (3.1)
YJM789	669, 329 (19.7)	452, 163 (12.1)	338, 105 (8.7)
YPS1009	189, 402 (10.2)	31, 88 (2)	3, 35 (0.7)
YPS163	186, 297 (8.4)	11, 41 (0.9)	

Strain	Expression Differences from S288c	FDR 0.05	
		Expression Differences from Mean	Expression Differences from YPS163
B1	178^a^, 260 b (7.3)	64, 33 (1.6)	126, 42 (2.8)
I14	177, 371 (9.1)	29, 39 (1.1)	32, 42 (1.2)
K1	288, 366 (11)	104, 57 (2.7)	101, 48 (2.5)
K9	414, 409 (13.8)	158, 93 (4.2)	166, 73 (4)
M22	360, 903 (24.7)	149, 437 (11.4)	120, 187 (6)
M8	240, 486 (12.3)	25, 112 (2.3)	36, 50 (1.5)
PR	266, 352 (10.6)	31, 98 (2.2)	34, 27 (1)
RM11-1a	667, 418 (21.4)	375, 206 (11.5)	288, 130 (8.3)
SB	219, 402 (10.7)	65, 87 (2.6)	97, 52 (2.6)
Y1	363, 309 (11.4)	189, 48 (4)	135, 38 (2.9)
Y10	237, 456 (11.8)	115, 57 (2.9)	95, 30 (2.1)
Y12	282, 303 (9.6)	58, 25 (1.4)	78, 24 (1.7)
YJM269	453, 428 (14.5)	247, 105 (5.8)	214, 101 (5.2)
YJM308	627, 568 (20.1)	264, 111 (6.3)	238, 76 (5.3)
YJM789	995, 555 (30.5)	774, 323 (21.6)	581, 201 (15.4)
YPS1009	365, 658 (17.6)	76, 192 (4.6)	18, 54 (1.2)
YPS163	322, 496 (14.3)	26, 82 (1.9)	

Expression differences relative to S288c, the mean of 17 strains, or strain YPS163 were defined at a false discovery rate (FDR) of 0.01 or 0.05. Values represent the number of genes expressed ^a^ higher or ^b^ lower than the designated reference. The percent of yeast genes affected in each strain at each significance threshold is shown in parentheses.

However, closer inspection revealed that many of these expression differences were common to all of the non-laboratory strains (Figure 3A), revealing that these expression patterns were unique to S288c. This group was enriched for functionally related genes, including those involved in ergosterol synthesis, mitochondrial function, respiration, cell wall synthesis, transposition, and other functions (Table 2). Many of these functional groups were also reported by Brem et al., who noted that multiple categories (including ergosterol synthesis and mitochondrial function) can be linked to a known polymorphism in the Hap1p transcription factor [44]. Indeed, the expression differences specific to S288c were enriched for targets of Hap1p (p<10^−11^, hypergeometric distribution) as well as targets of Hap4p (p<10^−6^) [45], which regulates genes involved in respiration. Hence, many of the observed expression differences may result because of S288c-specific physiology (see Discussion).

**Table 2:** Functional enrichment in genes differentially expressed in S288c.

Higher expression in S288c		*p* value
Phosphate metabolism a	7/33 b	1×10^−06^
Cell wall	6/38	3×10^−05^
Cytokinesis	4/5	4×10^−07^
Transposable element genes	71/90	1×10^−80^
Extracellular proteins	11/84	1×10^−07^
HELICc Domain	14/77	2×10^−11^
DEXDc Domain	13/80	5×10^−10^
**Lower expression in S288c**		
Respiration	20/88	1×10^−18^
Mitochondrion	35/366	1×10^−19^
Carbon Utilization	78/220	1×10^−103^
Sterol biosynthesis	10/25	6×10^−13^

Functional enrichment was calculated using the hypergeometric distribution with Bonferroni correction in the program FunSpec [82] on genes called differentially expressed (FDR 0.05) in 70% of all strains compared to S288c.

a Functional group with statistically significant enrichment.

b Number of genes in selected group compared to total number of genes in the genome with that annotation.

For a more representative description of expression variation in non-laboratory strains, we sought to represent the expression differences in a way that was not obscured by S288c. First, we identified genes whose expression varied significantly from the oak strain YPS163. Second, we identified transcripts whose abundance varied from the mean of all non-laboratory strains (see Materials and Methods). Although the mean expression value of each gene is merely an arbitrary reference point, this data transformation serves to remove the effect of S288c from each array while maintaining the statistical power to identify expression differences.

Roughly 1330 (23%) of yeast genes varied in expression in at least one non-laboratory strain relative to the mean of all strains, while 953 (17%) of genes varied significantly from YPS163 (FDR = 0.01). In both cases, two thirds of significant expression differences were specific to only one strain (Figure 3B and 3C). The number of genes with statistically significant expression differences from the mean ranged from 30 (in vineyard strain I14) to nearly 600 (in clinical isolate YJM789), with a median of 88 expression differences per strain. The number of expression differences did not correlate strongly with the genetic distances of the strains (R^2^ = 0.16). However, this is not surprising since many of the observed expression differences are likely linked in *trans* to the same genetic loci [27],[31],[34],[35],[43]. Consistent with this interpretation, we found that the genes affected in each strain were enriched for specific functional categories (Table S4), revealing that altered expression of pathways of genes was a common occurrence in our study.

We noticed that some functional categories were repeatedly affected in different strains. To further explore this, we identified individual genes whose expression differed from the mean in at least 3 of the 17 non-laboratory strains. This group of 219 genes was strongly enriched for genes involved in amino acid metabolism (p<10^−14^), sulfur metabolism (p<10^−14^), and transposition (p<10^−47^), revealing that genes involved in these functions had a higher frequency of expression variation. Differential expression of some of these categories was also observed for a different set of vineyard strains [26],[28], and the genetic basis for differential expression of amino acid biosynthetic genes in one vineyard strain has recently been linked to a polymorphism in an amino acid sensory protein [35]. We also noted that the 1330 genes with statistically variable expression in at least one non-laboratory strain were enriched for genes that contained upstream TATA elements [46] (p = 10^−16^) and genes with paralogs (p = 10^−6^) but under-enriched for essential genes [47] (p = 10^−25^). The trends and statistical significance were similar using 953 genes that varied significantly from YPS163. Thus, genes with specific functional and regulatory features are more likely to vary in expression under the conditions examined here, consistent with reports of other recent studies [30],[43],[48],[49] (seeDiscussion).

### Influence of Copy Number Variation on Gene Expression Variation

Expression from transposable Ty elements was highly variable across strains. However, Ty copy number is known to vary widely in different genetic backgrounds [50],[51], suggesting that these and other observed expression differences could be due to copy number variations in particular strains. Indeed, numerous expression differences could be linked to known gene amplifications in S288c, such as *ASP3*, *ENA1*, *CUP1*, and hexose transporters [52],[51]. We quantified the contribution of increased copy number to the observed increases in gene expression relative to S288c in 6 of our strains. In general, ∼2–5% of expression differences could be wholly or partially explained by differences in gene copy number (see Materials and Methods). YPS1009 was an exception to the trend, since nearly 20% of genes with higher expression could be attributed to increased copy number - most of these genes reside on Chromosome XII. In fact, more than 80% of genes on Chromosome XII met our criteria for increased copy number (Figure S1A), indicating that the entire chromosome is duplicated in this strain. Another example of chromosomal aneuploidy is evident in strain K9, for which Chromosome IX appears amplified (Figure S1B). Whole-chromosome aneuploidy has been frequently observed in strains growing under severe selective pressure (for example [53]–[56]. Interestingly, the majority of genes on these duplicated chromosomes do not show elevated transcript abundance in the respective strains.

In fact, only ∼25% of genes with increased copy number in each strain showed elevated expression (defined at FDR = 0.01 or as genes whose expression is >1.5× over S288c). This is in stark contrast to previous studies demonstrating little dosage compensation in S288c in response to gene amplification and chromosomal aneuploidy, leading to the conclusion that yeast does not have a mechanism for dosage compensation. [53],[54],[57]. Instead, our results suggest that some form of feedback control acts to normalize the dosage of most genes in non-laboratory yeast strains. The remaining quarter of amplified genes may be inherently exempt from this feedback mechanism. Alternatively, relaxed feedback may occur for specific amplifications if the resulting transcript increase provides a selective advantage to the strain in question. Indeed, 15–40% (depending on the strain) of genes lacking feedback control show at least 1.5× higher expression beyond what can be accounted for by gene amplification alone, indicating that the expression differences are affected by both gene dosage and regulatory variation. These genes are excellent candidates for future studies of adaptive changes.

As observed for gene expression, we found that some genomic amplifications were common across all 6 strains compared to S288c. All strains showed decreased Ty1 copy number, ranging from 2–15× lower than S288c. This is consistent with previous studies that showed higher Ty1 copy number (including active and partial Ty elements) in S288c compared to wine strains and natural isolates [50],[51],[58]. Most strains also showed even lower Ty1 transcript abundance, beyond what could be explained by copy number variations. Thus, in addition to a higher Ty content, S288c also shows higher expression from Ty genes, perhaps reflecting elevated rates of retrotransposition under the conditions studied here. In contrast, all strains showed higher copy number of the mitochondrial genome compared to S288c, typically elevated 2–3× but nearly 7× higher in clinical strain YJM789. The most likely explanation is that these strains harbor more mitochondria than S288c, a fact confirmed in vineyard strain RM11-1a by mitochondrial staining [25].

### Correlations between Altered Gene Expression and Environmental Sensitivity

In addition to revealing phenotypic diversity within and between yeast populations, natural variation can also uncover new insights into the effects of each environment on cellular physiology. For example, we noted correlations between environments based on the distribution of strain-sensitivity scores. The most likely explanation is that these stresses have similar effects on cellular function, and thus strains display similar sensitivities to them. Resistance to sodium chloride and lithium chloride or tolerance of ethanol and elevated temperature were highly correlated (R = 0.66 at p<0.0001 and R = 0.51 at p<0.0006, respectively, based on 10,000 permutations), consistent with the known effects of these stress pairs on ion concentrations or membrane fluidity/protein structure, respectively. Other relationships were not previously known, including the correlation between sensitivity to SDS detergent and the heavy metal cadmium (R = 0.64, p<0.0001) and between ethanol and caffeine tolerance (R = 0.59, p<0.0001). In contrast, resistance to freeze-thaw stress was anticorrelated to sodium chloride resistance (R = −0.35, p = 0.006), suggesting antagonistic outcomes of the same underlying physiology. These relationships point to commonalities in the cellular consequences inflicted by these environments that will be the subject of future investigations of stress-defense mechanisms.

We also conducted an associative study to identify gene expression patterns correlated with environmental sensitivity across the 17 non-laboratory strains (see Materials and Methods for details). As basal expression differences could significantly contribute to the inherent ability of cells to survive a sudden dose of stress, the results point to genes whose expression is related to, and perhaps causes, the phenotypes in question. Among the top genes associated with copper sulfate resistance was the metallotheionein *CUP1*, important for copper resistance and known to have undergone tandem duplications in copper-resistant strains [59],[60]. Of the genes whose expression was correlated to sodium chloride tolerance, nearly 20% are known to function in Na+ homeostasis and/or osmolarity maintenance (including *RHR2*, *COS3*, *SIS2* identified through genetic studies [61]–[63] and *JHD2*, *SRO7*, *YML079W*, *YOL159C*, *TPO4*, *UTH1* implicated in high-throughput fitness experiments in S288c [64]). Thus, these and likely other genes whose expression is highly correlated with each stress-sensitivity profile play a functional role in surviving that condition.

Other correlations were not expected. Ethanol and caffeine tolerance were both correlated to the expression of genes encoding transmembrane proteins (p<0.003, hypergeometric distribution), perhaps related to the effect of these drugs on membrane fluidity. Sensitivity to the cell-wall damaging drug Congo Red was significantly correlated to the expression of genes involved in mitochondrial function and translation, respiration, and ATP synthesis (p<10^−13^), revealing a link between mitochondria/respiration and the cell wall. Although these connections will require further characterization, they demonstrate the power of using natural diversity to uncover previously unknown relationships between stresses and cellular processes.

## Discussion

This study demonstrates the vast amount of phenotypic variation in *Saccharomyces* strains collected from diverse natural habitats, used in industrial processes, and associated with human illness. Considering the phenotypic responses to the conditions studied here provides insights into the relationships between specific strains and their niches. For example, the wide variance in growth scores of sake-producing strains indicates that they are either highly resistant or sensitive to the different environments studied here, suggesting that they may be specialized for growth in the defined conditions of sake fermentation. In contrast, many of the vineyard isolates survived relatively well in most of the conditions tested. This may reflect their ability to thrive in more variable, natural environments and may also have facilitated their dispersal into new environments in a manner associated with human interactions [5]. Geographic dispersal might also explain the higher-than-expected phenotypic diversity of vineyard strains, which might be driven by diversifying selection (suggested by our analysis) due to unique pressures imposed after expansion into new environments.

Although many of the phenotypic differences we observed are probably neutral, providing no benefit or disadvantage to the strains in question, some are likely to provide a selective advantage. Copper-sulfate resistance in European vineyard strains may have arisen through positive selection, since copper has long been used as an antimicrobial agent in vineyards and orchards [1],[65]. Another example may apply to the oak strains studied here. Our simple metric comparing phenotypic to genetic diversity in strains collected from similar environments suggests that oak strains are phenotypically more similar than expected based on their genetic relationship. Formally, this could arise if multiple traits are evolving neutrally (but slower than the genetic drift represented by the sequences used here) since the strains diverged from a distant, common progenitor. However, the fact that *S. paradoxus* oak isolate YPS125 is phenotypically more similar to *S. cerevisiae* oak strains than the other *S. paradoxus* isolate in our analysis instead supports that these strains have undergone selection for growth in a common environment. One intriguing phenotype is freeze-thaw resistance, which may be important to survive the wintry niche from where these strains were collected. Consistent with this hypothesis, we have recently isolated numerous *Sacharomycete* strains (including *S. cerevisiae*) from Wisconsin oak exudates, of which 86% (19/22) are freeze-thaw tolerant (DJK and APG, unpublished data). Ongoing studies in our lab are dissecting the genetic basis for this phenotypic difference.

In addition to stress sensitivity, gene expression also varies significantly across yeast populations. More than a quarter of yeast genes varied in expression in at least one non-laboratory strain under the conditions studied here. Consistent with other recent reports [30],[48],[49],[66], we find that genes with specific structural or functional characteristics (including nonessential genes and those with upstream TATA elements and paralogs) show higher levels of expression variation across strains. This has previously been interpreted as a higher rate of regulatory divergence for genes with these features, either in response to selection [48] or mutation accumulation [49]. However, these features are also common to genes whose expression is highly variable within the S288c lab strain grown under different conditions ([67] and data not shown), particularly those induced by stressful conditions [46],[68]. It is also notable that genes with TATA elements show higher ‘noise’ in gene expression within cultures of the same strain [69],[70]. Thus, an alternative, but not necessarily mutually exclusive, hypothesis is that the expression of these genes is more responsive to environmental or genetic perturbations, again consistent with previous studies [66],[30],[48],[49]. We have conducted our experiments under ‘common garden’ lab conditions in attempt to minimize environmental contributions to expression phenotypes. However, because each strain may have evolved for growth in a unique environment, each may in fact respond differently to the same growth conditions used here. Indeed, this may explain the prevalence of metabolic genes in our set of genes showing variable expression in multiple strains, since many of these strains have not evolved for growth in highly artificial laboratory media.

Emerging from our analysis is the fact that S288c is phenotypically distinct from the other non-laboratory strains studied here. This strain displays extreme resistance to specific stresses, harbors fewer mitochondria, contains more transposable elements, and shows unique expression of many genes compared to all other strains investigated (a direct comparison of the number of differentially expressed genes in S288c is difficult due to the different statistical power in calling these genes). We have also found that this strain has an aberrant response to ethanol, since it is unable to acquire alcohol tolerance after a mild ethanol pretreatment, unlike natural strains [71]. It is likely that additional responses found in natural strains have been lost or altered in this domesticated line. The progenitor of S288c was originally isolated from a fallen fig in Merced, California, and sequence analysis indicates that S288c is genetically similar to other natural isolates [1]–[3]. A recent study by Ronald et al. counters the proposal that S288c has undergone accelerated divergence during its time in the laboratory [72]. Instead, our results suggest that the strain has evolved unique characteristics through inadvertent selection for specific traits (such as growth on artificial media) and population bottlenecks. Thus, the laboratory strain of yeast may not present an accurate depiction of natural yeast physiology. Indeed, no single strain can be used to accurately represent the species, a note especially important for comparing phenotypes across species. Complete exploration of an organism's biology necessitates the study of multiple genetic backgrounds to survey physiology across populations.

Despite its limitations, the lab strain offers nearly a century of detailed characterization, along with powerful genetic and genomic tools. A useful approach is to complement studies on laboratory strains with investigations of natural variation. By characterizing stress sensitivity in a large set of strains, we have leveraged the power of natural diversity to uncover new relationships between stresses and to reveal previously unknown connections between genes, stresses, and cellular processes. These connections lead to hypotheses about stress defense mechanisms that can often be dissected using the valuable tools provided by the lab strain. Application of genomic techniques to characterize natural yeast strains will foster such studies while revealing additional insights into genetic and phenotypic variation in *Saccharomyces*.

## Materials and Methods

### Strains and Sequence Analysis

Strains used in this study and references are found in Table S1. In addition to sequence data from [2], an additional 5,305 bp of noncoding DNA was sequenced for 41 *S. cerevisiae* strains over 8 intergenic sequences (GENBANK accession numbers EU845779 - EU846095) for a total of 13,016 bp over 13 loci. Phylogenetic analysis shown in Figure 1 was performed on the combined sequence set using the program MrBayes [73]. Evolutionary distances were estimated using the Jukes-Cantor (JC) model based on 2,056 bp noncoding sequence data present in all strains; results and significance were very similar when the distance was based on 9,334 bp of noncoding sequence excluding only pairwise-deletion data [74]. Strains with evolutionary distances equal to zero over this subsequence (but clearly non-zero when all sequence was assayed) were set to 0.00001 to facilitate permutation calculations. Paralogs were defined as genes with a BLAST E-value score <10^−100^.

### Phenotyping and Analysis

Yeast strains were grown in YPD medium at 30°C to an optical density of ∼0.3 in 96-well plates. Three 10-fold serial dilutions were spotted onto YPD agar plates containing the appropriate stress, as well as a YPD plate for a no-stress control. Cells were also plated onto minimal medium [75] or YP-acetate. In the case of freeze-thaw stress, 200 µl cells was frozen in a dry ice/ethanol bath for two hours or left on ice as a control before spotting onto YPD plates. Cells were grown for 2–3 days at 30°C unless otherwise noted, and viability of each dilution was scored relative to the no-stress control for each strain. All experiments were done in at least duplicate over 2–3 doses of most stresses (see Table S2 for raw data and stress doses). Final resistance scores were summed over the 3 serial dilutions then averaged over replicates and stress doses, providing a single score ranging from 0 (no growth) to 6 (complete growth) for each strain and each stress condition.

For Figure 2, strains were clustered based on phenotypes using the Pearson correlation and UPGMA clustering [76]. Correlations between stresses were calculated based on the Pearson correlation between strains, excluding 14 strains of highly similar genetic distance (JC<0.0008). Phenotypes specific to groups of strains collected from similar environments (see Table S1 for groupings) were calculated based on the median growth score of strains in that group. Significance was estimated by 10,000 permutations of strain-group labels, scoring the frequency of observing a median growth score equal to or greater than that observed.

A parameter, P/G, was calculated to compare the similarity in phenotype to the similarity in genotype for strains within and between niche groups. The average pairwise phenotypic distance, taken as the Pearson distance (1 – Pearson correlation) between phenotype vectors, was divided by the average pairwise JC distance for strains within a niche group. This value was divided by the same ratio calculated for all pairs of strains between niche groups (see Table S1 for niche groupings). Significance was estimated based on 10,000 random permutations of strain-group labels. The distribution of P/G ratios from randomized trials was centered on 0.99; furthermore P/G was ∼1.0 for strains in the ‘clinical’, ‘natural’, and ‘other fermentation’ groups, reflecting either neutral drift for these groups or that these strains were inappropriately grouped together into somewhat amorphous categories.

### Gene Expression Analysis

Seventeen strains (including B1, I14, M22, M8, PR, RM11-1a, K1, K9, YJM308, YJM789, YJM269, Y12, SB, Y1, Y10, YPS1009, and YPS163) were chosen for whole-genome expression analysis. Cells were grown 2–3 doublings in YPD medium to early log-phase in at least biological triplicate. Cell collection, RNA isolation, and microarray labeling and scanning were done as previously described [77], using cyanine dyes (Flownamics, Madison, WI) and spotted DNA microarrays consisting of 70mer oligos representing each yeast ORF (Qiagen). For all arrays, RNA collected from the denoted strain was compared directly to that collected from the diploid S288c lab strain DBY8268, with inverse dye labeling used in replicates to control for dye-specific effects. At least three biological replicates were performed for all comparisons. Data were filtered (retaining unflagged spots with R^2^>0.1) and normalized by regional mean-centering [78]. Genes with significant expression differences (compared to the S288c control, strain YPS163, or the mean expression across all strains) were identified separately for each strain with a paired t-test (or unpaired t-test in reference to YPS163) using the BioConductor package Limma v. 2.9.8 [79] and FDR correction [80], taking p<0.01 as significant unless otherwise noted (see Table S3 for limma output and Figure S2 for a comparison of the statistical power for each strain). All microarray data are available through the NIH Gene Expression Omnibus (GEO) database under accession number GSE10269.

### Comparative Genomic Hybridizations

Array-based comparative genomic hybridization (aCGH) was performed in duplicate on six strains (K9, M22, RM11-1a, Y10, YJM789, and YPS1009) relative to the DBY8268 control as previously described [81], using amino-allyl dUTP (Ambion), Klenow exo-polymerase (New England Biolabs), and random hexamers. Post-synthesis coupling to cyanine dyes (Flownamics) was performed using inverse dye labeling in replicate experiments. Technical variation in hybridization was defined as the mean+2 standard deviations (a log2 value of 0.3) of all spot ratios, based on triplicate comparisons of DBY8268 to DBY8268 genomic DNA. For non-lab strains compared to DBY8268, genes with negative aCGH ratios outside the range of technical variation on both duplicates were defined as those affected by copy number and/or hybridization defects. Transcript levels within 0.45 (3 standard deviations of technical variation) of the aCGH ratio were identified as those largely explained by copy number and/or hybridization defects – on average, fewer than 5% of genes with statistically significant (FDR = 0.01) differential expression compared to DBY8268 fell into this class. Genes with a positive aCGH ratio >0.7 in log2 space were defined as genes with increased copy number in each non-lab strain. All microarray data are available through the NIH Gene Expression Omnibus (GEO) database under accession number GSE10269.

### Associations between Phenotype and Gene Expression Vectors

A vector of relative phenotype scores was generated by dividing scores from Figure 2 by the score measured for DBY8268. The Pearson correlation between this vector and the measured expression vector for each strain relative to DBY8268 was calculated for all genes in the dataset. Genes whose expression was correlated above or below what was expected by chance (*p*<0.01) were defined based on 100 permutations of each of the ∼6,000 expression vectors.

## Supporting Information

Figure S1 Chromosomal aneuploidy in specific *S. cerevisiae* strains. Log2 ratios of copy number variations in (A) YPS1009 and (B) K9 compared to S288c are shown for each of the 16 yeast chromosomes. Each red bar indicates an elevated aCGH ratio measured at a given yeast gene, while each green bar indicates a decreased aCGH ratio compared to S288c. The height of each bar is proportional to the aCGH ratio measured on the arrays and represents the average of duplicate hybridizations.
(1.04 MB TIF)

Figure S2 GEL50 plots representing statistical power. The fraction of genes called statistically significant at FDR 0.01 is plotted against the log2 value of relative gene expression. Genes were binned over 0.3 increments in gene expression and smoothed using a running average over 3 adjacent bins. The median GEL50, the log2 value at which 50% of measurements were called statistically significant, was 1.4.
(0.68 MB TIF)

Table S1 Strains used in this study.
(0.03 MB XLS)

Table S2 Raw phenotype scores, conditions, and stress doses used to make Figure 2.
(0.05 MB XLS)

Table S3 limma output for uncentered and mean-centered expression data.
(8.22 MB ZIP)

Table S4 Functional GO enrichment.
(0.20 MB TXT)
