# Supplementary figures and images for: Variations in Stress Sensitivity and Genomic Expression in Diverse S. cerevisiae Isolates
Source: PLoS Genet. 2008 Oct 17;4(10):e1000223. doi: 10.1371/journal.pgen.1000223 (PMC2562515; doi:10.1371/journal.pgen.1000223)

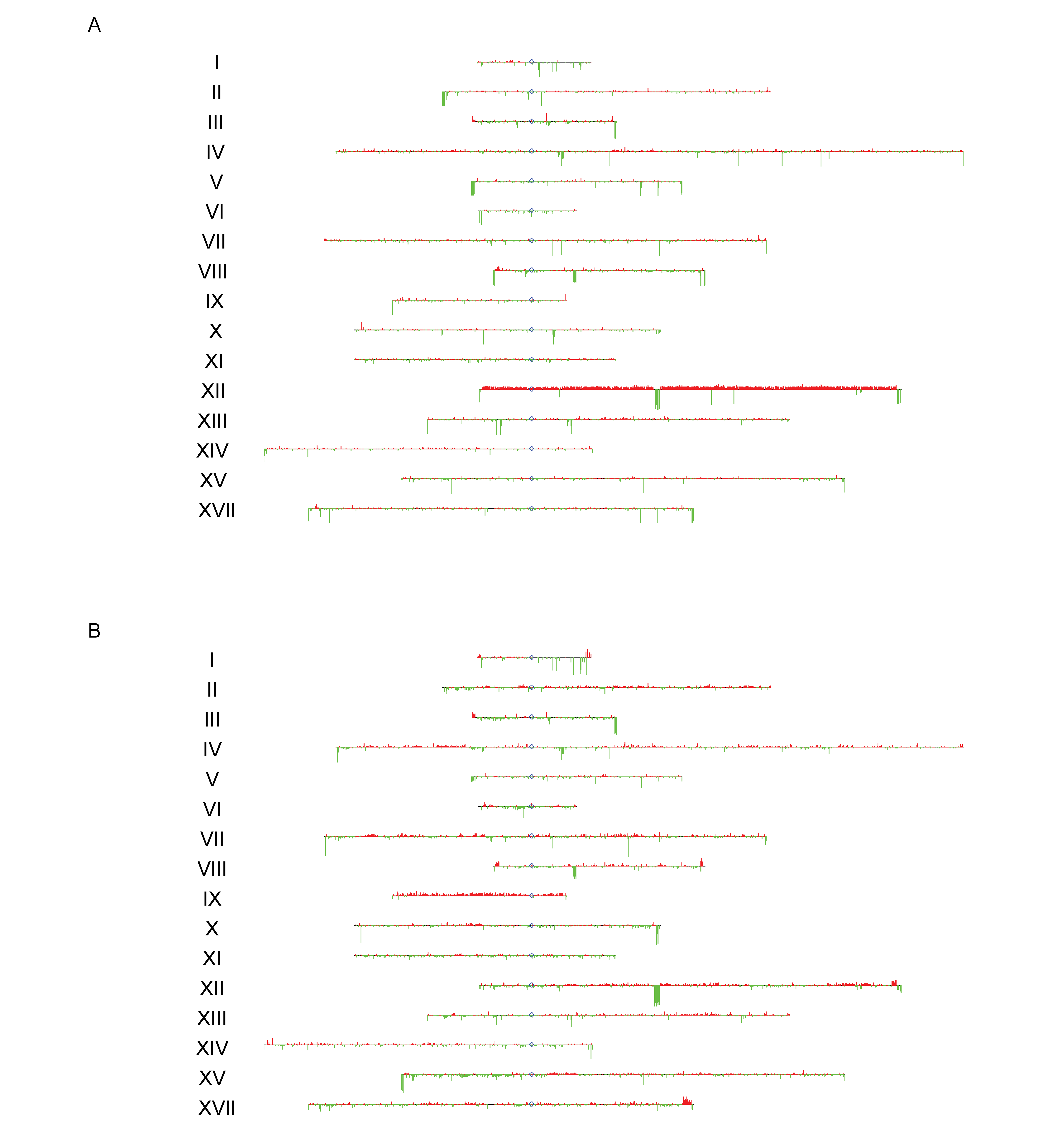

Supplement: Figure S1 — Chromosomal aneuploidy in specific S. cerevisiae strains. Log2 ratios of copy number variations in (A) YPS1009 and (B) K9 compared to S288c are shown for each of the 16 yeast chromosomes. Each red bar indicates an elevated aCGH ratio measured at a given yeast gene, while each green bar indicates a decreased aCGH ratio compared to S288c. The height of each bar is proportional to the aCGH ratio measured on the arrays and represents the average of duplicate hybridizations. (1.04 MB TIF) [file pgen.1000223.s001.tif]

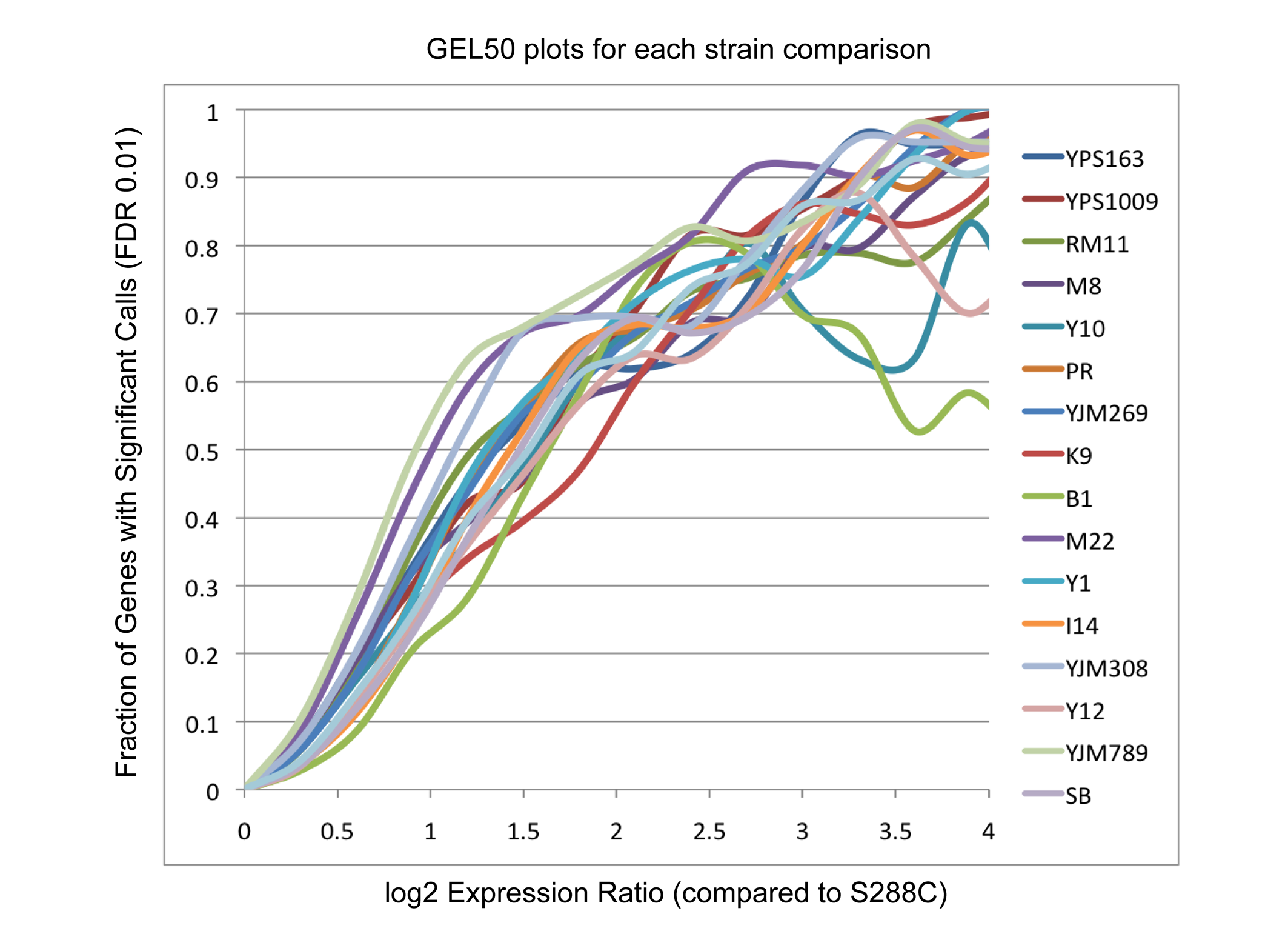

Supplement: Figure S2 — GEL50 plots representing statistical power. The fraction of genes called statistically significant at FDR 0.01 is plotted against the log2 value of relative gene expression. Genes were binned over 0.3 increments in gene expression and smoothed using a running average over 3 adjacent bins. The median GEL50, the log2 value at which 50% of measurements were called statistically significant, was 1.4. (0.68 MB TIF) [file pgen.1000223.s002.tif]
